# Serum cholinesterase activity as an early indicator of systemic inflammation

**DOI:** 10.1186/cc14132

**Published:** 2015-03-16

**Authors:** AR Zivkovic, K Schmidt, J Bender, T Brenner, S Hofer

**Affiliations:** 1 Heidelberg University Hospital, Heidelberg, Germany

## Introduction

Early diagnosis of systemic inflammation, a generalised response to noxious stimuli, is fundamentally important for effective and goal-directed therapy. Various inflammation biomarkers have been used in clinical and experimental practice. However, a definitive diagnostic tool for an early detection of systemic inflammation remains to be identified. Acetylcholine (Ach) has been shown to play an important role in the inflammatory response. Serum cholinesterase (butyrylcholinesterase (BChE)) is the major Ach hydrolyzing enzyme in plasma. The role of this enzyme during inflammation has not yet been fully understood. Here, we describe a correlation between the BChE activity and the early systemic inflammatory response upon traumatic injury.

## Methods

We measured BChE activity in patients with traumatic injury admitted to the emergency room using a point-of-care-test (POCT) system. In addition, we measured levels of routine inflammation biomarkers during the initial treatment period. We used the Injury Severity Score to assess the trauma severity. Data were statistically analyzed using the Friedman test. Correlation analysis was performed using Spearman's rank correlation test. *P *< 0.05 was considered statistically significant.

## Results

Reduced BChE activity correlated with trauma severity and the resulting systemic inflammation. Compared with serum levels of routinely measured inflammatory biomarkers, changes in the BChE activity were detected significantly earlier, suggesting that the BChE activity might serve as an early indicator of systemic inflammation.

## Conclusion

Our results suggest that BChE activity, measured using a POCT system, might play an important role in the early diagnosis of trauma-induced systemic inflammation.

